# A Case of Native Valve Infective Endocarditis and Bacteremia Due to Streptococcus cristatus

**DOI:** 10.7759/cureus.67122

**Published:** 2024-08-18

**Authors:** Ana Sofia Silva, Mariana Baptista, Inês Soares, Diogo Macedo, Rita R Neto

**Affiliations:** 1 Internal Medicine, Unidade Local de Saúde Gaia e Espinho, Vila Nova de Gaia, PRT

**Keywords:** endocarditis, bacteremia, infective endocarditis, s. cristatus, streptococcus cristatus

## Abstract

Infective endocarditis is a severe infection affecting the inner lining of the heart chambers and valves, often preceded by bacteremia and frequently caused by organisms from the commensal oral flora, including *Streptococcus (S.) cristatus*. However, *S. cristatus* remains an uncommon causative agent.

We present the case of a 68-year-old male with a history of dyslipidemia, severe aortic insufficiency, and mild mitral insufficiency who presented with a two-week history of fever without other symptoms. Blood cultures were positive for *Streptococcus cristatus*, and a transthoracic echocardiogram revealed multiple vegetations on the aortic valve and smaller vegetations on the mitral valve. Additionally, splenic embolization was detected. The patient was treated with benzylpenicillin and gentamicin, followed by aortic valve replacement surgery.

This case highlights a previously immunocompetent patient who developed bacteremia and fulfilled the definitive criteria for infective endocarditis, characterized by severe aortic valve insufficiency and splenic embolization, caused by a commensal oral bacterium rarely reported in clinical cases. The severity of the infection necessitated surgical intervention, and the patient subsequently made a full recovery without major complications post-discharge.

## Introduction

*Streptococcus cristatus* (*S. cristatus*) is a gram-positive coccus commonly found in the human oral microbiome and able to cause biofilms. It belongs to the Mitis group of the Streptococcus genus. This bacterium is considered a commensal organism and, normally, is not a cause of disease [1]. It was first characterized in 1991, as phenetic tests became more sophisticated [1].

Despite its commensal nature, *S. cristatus* has emerged as a causative agent in various human infections, including dental abscesses, periodontitis, endocarditis, osteomyelitis, endophthalmitis, and septic arthritis [2-6]. Recent reports indicate that *S. cristatus* can lead to infective endocarditis (IE) with significant complications, even in individuals without underlying health conditions [2,5,6].

## Case presentation

A 68-year-old male with a medical history of dyslipidemia treated with rosuvastatin and benign prostatic hyperplasia treated with finasteride and tamsulosin.

Six months before this episode, normocytic and normochromic anemia (with a hemoglobin of 9.8 g/dL, normal values 13.0-18.0 g/dL for men) was detected in routine analysis. The workup investigation included an endoscopic study for which the patient had to perform an echocardiography, which identified an asymptomatic severe aortic insufficiency and mild mitral insufficiency. The patient had no history of previous dental procedures or poor oral hygiene, as well as toxiphilic habits.

He presented to the Emergency department with a hemoglobin level of 7.6 g/dL, after being referred from the clinic where he underwent an endoscopic evaluation. Upon admission, a fever was documented, with an ear temperature of 39 °C. The patient reported having had a fever for approximately two weeks, which he had not considered significant. He denied any other symptoms or focal complaints, including respiratory, cardiovascular, urinary, or abdominal issues. During the physical examination, there were no signs or symptoms of acute heart failure, including new cardiac murmurs, pulmonary congestion, or pitting edema. Additionally, no skin manifestations indicative of infective endocarditis (IE), such as Roth's spots, Osler's nodes, or Janeway lesions, were observed.

The initial workup revealed normocytic and normochromic anemia without leukocytosis or alterations in platelet count. The patient had an erythrocyte sedimentation rate (ESR) of 90 mm/h (normal <20 mm/h) but no elevation in C-reactive protein. No other significant laboratory abnormalities were identified.

An electrocardiogram and a chest X-ray were also performed, both of which showed no significant abnormalities. Given the patient's persistent fever, absence of focal symptoms, and recent findings of valvular insufficiency, he was admitted and started on empirical antibiotic therapy with ceftriaxone 2 g daily, under the suspicion of possible IE.

Blood cultures were taken, and the patient was admitted for further evaluation. Two days later, *Streptococcus cristatus* was identified in all four blood cultures (MIC 0.25-1 μg/mL for Benzylpenicillin). Based on the likely diagnosis of infective endocarditis caused by *S. cristatus*, we initiated targeted antibiotic therapy with Benzylpenicillin (4 M.U. IV every 4 hours) and gentamicin (230 mg IV daily), according to the recommendations.

**Figure 1:**
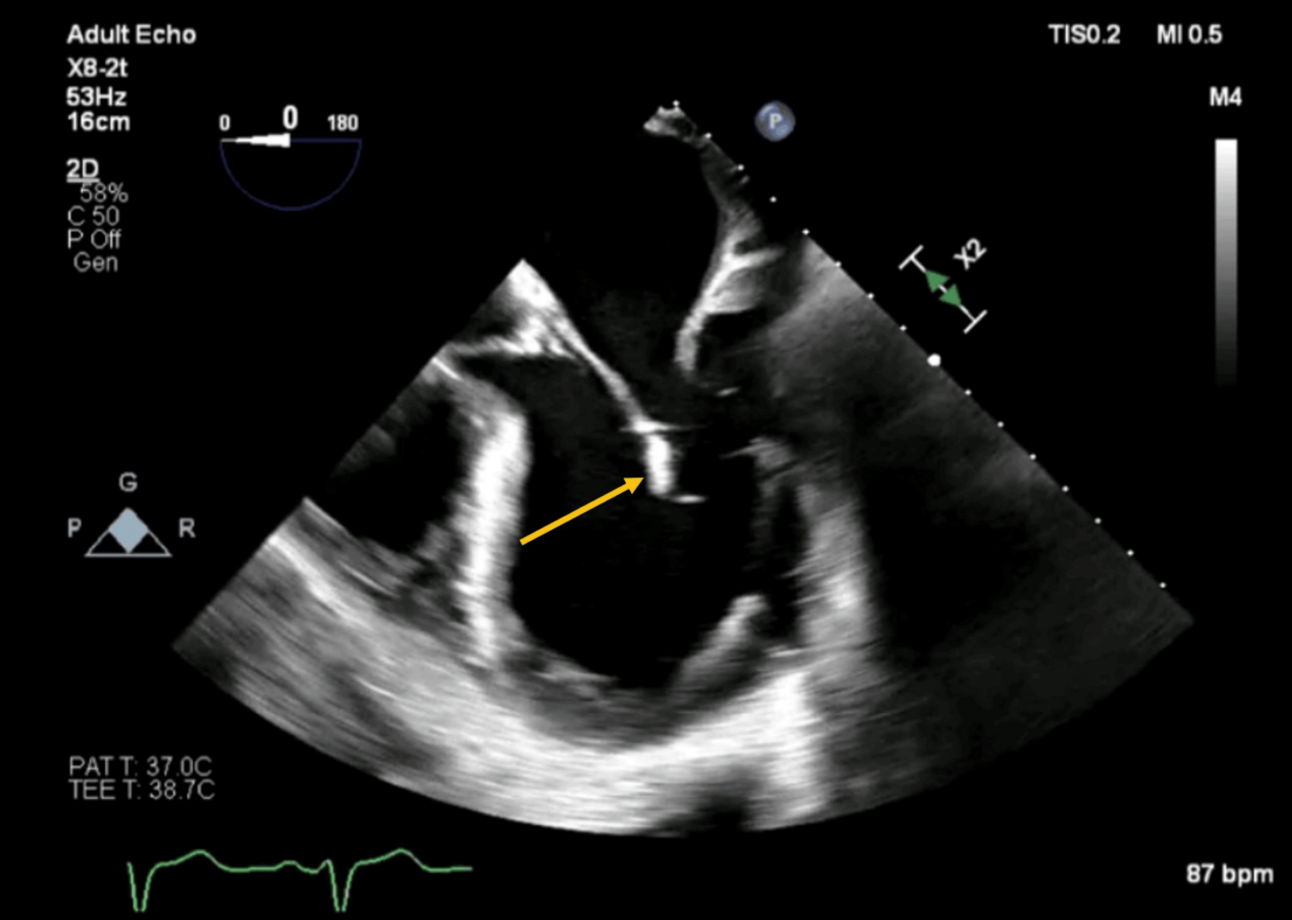
Vegetations in transesophageal echocardiogram

**Figure 2:**
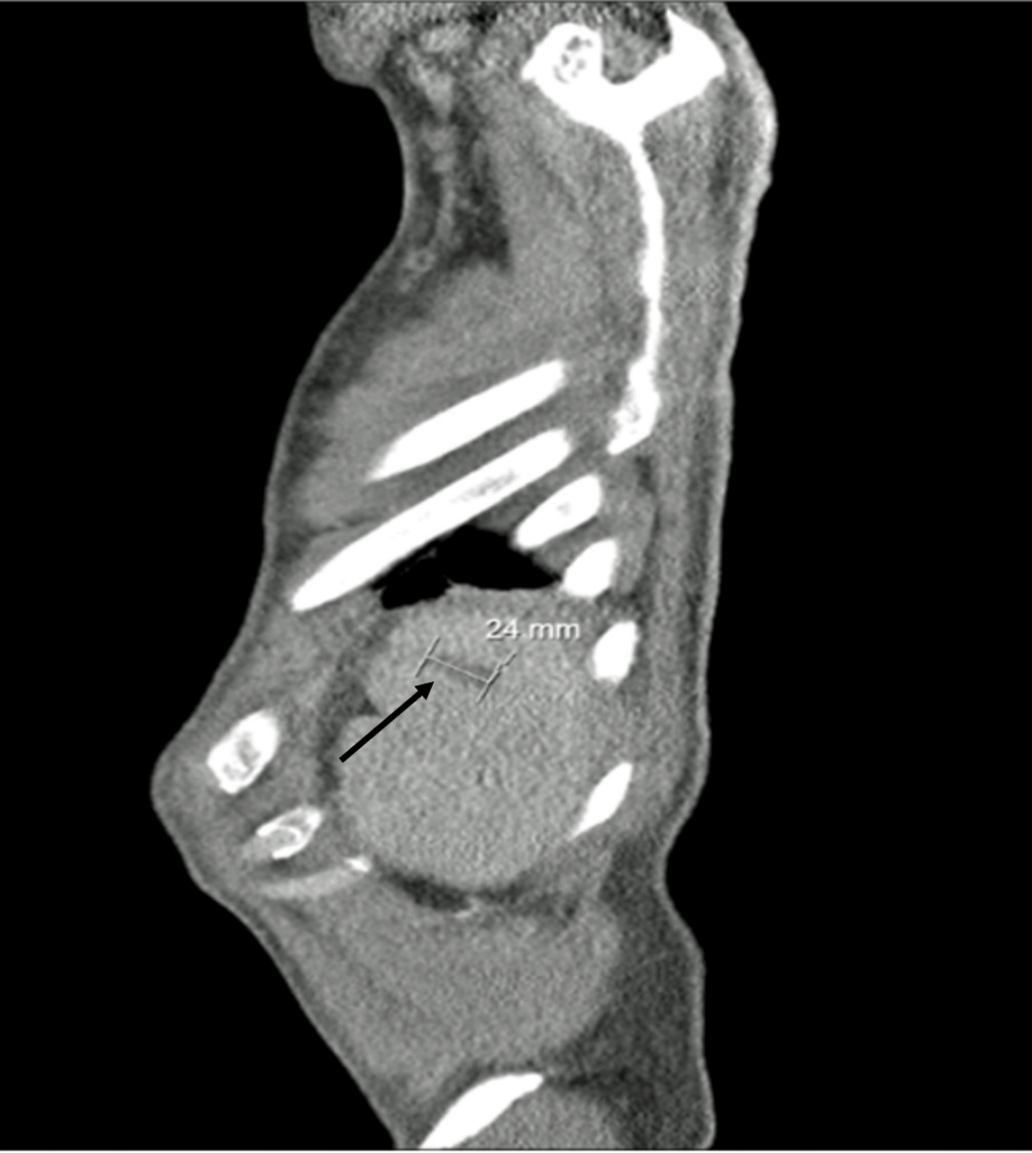
Septic spleen embolization

A transthoracic echocardiogram revealed multiple vegetations on the aortic valve, the largest measuring 22x17 mm, along with the previously known severe aortic insufficiency, which was confirmed by a transesophageal echocardiogram (Figure 1). The echocardiogram also suggested vegetations on the mitral valve, associated with mild insufficiency. An abdominal ultrasound identified a small splenic infarction, likely due to septic embolization (Figure 2).

These findings met the definite diagnosis of IE, according to the Modified Duke Criteria (two major criteria and one minor criterion).

The patient was also evaluated by the Stomatology department, which identified two lesions, one of which was necrotic, associated with chronic periodontitis. The two infected teeth were subsequently extracted.

He completed three weeks of targeted antibiotic therapy before undergoing surgery, which involved aortic valve replacement and removal of mitral valve vegetations. He then completed a six-week course of antibiotic therapy following the clearance of blood cultures. Both the surgical intervention and antibiotic therapy were successful, with negative cultures from intraoperative material and blood samples, and no new complications arose during the patient's follow-up, namely, new-onset heart failure, prosthetic dysfunction, local or systemic infection, recurrence of endocarditis, or hemorrhagic complications such as hematomas or massive bleeding.

The patient is presently fully independent, with complete restoration of his prior functional status and no evidence of heart failure symptoms. Consequently, he was discharged from follow-up care in both the Cardiothoracic Surgery and Internal Medicine clinics.

## Discussion

*Streptococcus cristatus* is still a rare agent of disseminated infection, possibly due to its commensal behavior and presence in the normal oral microbiota [1]. According to our research, this is the thirteenth clinical case of infection by *S. cristatus* and the second of invasive bacteremia. The other reports involved cases of bacteremia, infective endocarditis, endophthalmitis, pneumonia, and septic arthritis and involved both pediatric and middle-aged patients [2-7].

Infective endocarditis is a potentially fatal infection of the heart valves and endocardium, which can occur even in immunocompetent and asymptomatic patients. The importance of early and accurate diagnosis in these patients cannot be overstated, as the disease can progress rapidly and lead to severe complications, including heart failure, septic embolism, and death. The initial presentation of endocarditis can be nonspecific, such as isolated fever without other cardinal signs. In such cases, careful clinical surveillance is essential. Persistent and unexplained fever, even in the absence of other evident symptoms, should raise the suspicion of endocarditis [8].

*S. cristatus* is a less common but relevant bacterium in the etiology of infective endocarditis. Oral assessment is crucial because many bacteria involved in infective endocarditis originate from the oral cavity. Dental procedures, inadequate oral hygiene, and periodontal diseases are potential entry points for this pathogen [1,5,7].

Cardiac valvular pathology, particularly in patients with severe valvular diseases, requires special attention to the prevention of infections that could lead to infective endocarditis. Pathogenic agents frequently involved include oral flora microorganisms, such as *S. viridans* and *S. cristatus*, which can enter the bloodstream through oral lesions or dental procedures via bacterial translocation. Preventive strategies include rigorous oral hygiene, regular dental check-ups, prophylactic antibiotics for invasive dental procedures, and periodic cardiac evaluations to assess valvular function and the presence of complications such as progressive valvular insufficiency or new vegetations [5,7].

Both transthoracic echocardiography (TTE) and transesophageal echocardiography (TEE) play a crucial role in the diagnosis of infectious endocarditis. TTE is a valuable initial tool due to its non-invasive nature and ability to quickly provide images of cardiac structures, identifying vegetations, abscesses, or other valvular anomalies. However, TEE offers superior sensitivity and specificity, especially in cases where smaller lesions or those located in regions difficult to visualize by TTE, such as the mitral valve or prosthetic valves, are suspected. The combination of these two echocardiographic methods allows for a comprehensive and precise evaluation, which is essential for early diagnosis and effective management of endocarditis, directly influencing prognosis and therapeutic strategies [9].

Utilizing these complementary examinations, we seek a range of specific findings that may indicate the presence of infectious endocarditis. These findings include vegetations, abscesses, signs of valve perforation or rupture, aneurysms, prosthetic valve dehiscence, pseudoaneurysms, cardiac fistulas, and valvulopathies. Any of these findings are critical for confirming the diagnosis of infectious endocarditis, assessing disease severity, and guiding therapeutic decisions, including the need for surgical intervention [9,10].

Infective endocarditis is a condition that often requires surgical intervention, especially in cases complicated by valvular dysfunction. The decision regarding the necessity of surgery and the optimal timing for its performance is based on various clinical and hemodynamic factors. The main indications for cardiac surgery include severe valvular dysfunction resulting from infection, which can lead to congestive heart failure refractory to medical treatment and cardiogenic shock [10].

The timing for surgery should be evaluated on a case-by-case basis and is particularly reserved for issues such as uncontrolled infections, defined as persistent bacteremia despite adequate antibiotic therapy for more than 7-10 days, the presence of perivalvular abscesses or cardiac fistulas, and infections caused by fungi or multidrug-resistant microorganisms where medical therapy is insufficient to eradicate the infection. Other indications include cases with recurrent embolic complications or large thrombi, vegetations larger than 10 mm associated with a high embolic risk, and severe valvular dysfunctions [10].

Due to the risks of emergent surgery, the patient received a period of antibiotic treatment and surveillance; it is important to highlight the vigilance of alterations of consciousness, vision, renal failure, and other features that might denote eventual embolization to other important organs, such as the brain, eye, kidney or liver, which can be irreversible [8,10,11].

The surgical treatment of infective endocarditis typically involves repairing or replacing heart valves that have been damaged by the infection. This is usually done in cases where antibiotic treatment alone is not sufficient to cure the infection or when the infection has caused significant damage to the heart valves. The surgical procedure may involve removing the infected tissue and replacing it with a prosthetic valve or repairing the existing valve. In this particular case, the patient received a complex surgery, replacing the aortic valve and removing the excessive vegetation in the mitral valve [8].

It is extremely important to complete the antibiotic therapy, preferably directed at the isolated agent, for the necessary time after negative blood cultures and/or the surgical procedure [8]. Similarly, it is crucial in the early postoperative days and during early follow-up to rule out complications associated with cardiac surgery, such as local infectious complications or recurrence of endocarditis, renal complications, respiratory issues such as pleural effusion or respiratory distress, arrhythmias or new-onset heart failure, prosthetic dysfunction, and hemorrhagic complications [8,10].

## Conclusions

This case underscores the association among infective endocarditis, bacteremia, and *S. cristatus*. The described patient not only manifested definite infective endocarditis but also severe aortic valve insufficiency and splenic embolization, attributed to a commensal oral bacterium with an elusive pathogenic course.

The present case contributes scientific evidence to the potential of *S. cristatus* to induce serious infections, inviting further research in this domain.
